# A new approach to prevent cervical stenosis in postmenopausal women after loop electrosurgical excision procedure: a randomized controlled trial

**DOI:** 10.1038/s41598-020-65170-2

**Published:** 2020-05-22

**Authors:** Jing Lin, Yu Meng, Yi Chen, Zhunan Li, Ying Xu, Dan Wu

**Affiliations:** 1 The International Peace Maternity and Child Health Hospital, School of Medicine, Shanghai Jiao Tong University, Shanghai, China; 2 Shanghai Municipal Key Clinical Specialty, Shanghai, China

**Keywords:** Cancer, Gynaecological cancer, Cervical cancer

## Abstract

To determine whether regular cervical dilatation is effective for preventing cervical stenosis, and to identify the associated risk factors, in postmenopausal women after LEEP. This was a prospective randomized clinical trial in postmenopausal women who underwent LEEP at our hospital between August 2018 and May 2019. Patients who met the study criteria were randomly allocated to three groups: control group (without any intervention), intervention group A (underwent cervical dilatation at the 3^rd^, 5^th^, and 8^th^ week after LEEP) and intervention group B (underwent cervical dilatation at the 4^th^, 8^th^, and 12^th^ week after LEEP). A colposcopic follow-up examination was conducted at 6 months after LEEP to determine the incidence of cervical stenosis. A total of 404 postmenopausal women were found to be finally eligible for the study. The rate of cervical stenosis in the control group was significantly higher than that in the intervention group, and the rate in group A was significantly lower than that in group B. We found regular dilatation after LEEP in postmenopausal women can prevent cervical stenosis. Further, the 3rd, 5th, and 8th weeks after LEEP are optimal time points. Finally, LEEP frequency and resection depth are significant risk factors and can be used to screen postmenopausal women at risk for cervical stenosis after LEEP.

## Introduction

In recent years, loop electrosurgical excision procedure (LEEP) has become a conventional surgical method for the treatment of cervical lesions. The convenience of this operation, its strong diagnostic power, and its high patient acceptance rate have made LEEP popular, but unfortunately, the adverse consequences of this procedure have not been well studied. As the number of women undergoing LEEP is dramatically increasing, it is important to pay attention to some of its complications. In particular, cervical stenosis, which is a long-term complication of LEEP, is often neglected, as few studies have addressed the risk assessment and prevention measures of cervical stenosis.

One of the main drawbacks in the diagnosis and prevention of post-LEEP cervical stenosis is that there are no clear or consistent criteria for defining it, as the term “cervical stenosis” has been used to describe various types of cervical conditions from the subjective impression of stenosis to complete occlusion of the cervical orifice^1^. Some of the reported criteria for the diagnosis of cervical stenosis are the inability to intracervical sample through the cervix by cell brushes^2^, inability to pass a 3-mm cervical curette through the cervical canal^3^, and inability to pass a 4-mm cotton swab^1,4^. These differences in definitions might explain the vast differences in the overall incidence of cervical stenosis, which ranges from 3.4% to 32.7%^2^, as well as the vast difference in the reported rates of cervical stenosis after LEEP^1^. Despite this, examining patients for cervical stenosis after LEEP is important, as patients are at risk of recurrent and residual cervical lesions even after satisfactory treatment. Cervical stenosis affects the efficiency of colposcopy examination during follow-up after surgery, thus affecting the risk of recurrent and residual cervical lesions^1^. The prevalence of cervical stenosis in CIN (cervical intraepithelial neoplasia) recurrences was statistically higher in postmenopausal women compared with perimenopausal ones. Postmenopausal women often have a high incidence of stenosis and no obvious clinical manifestation, and hence, it is easy to miss this condition. Besides, there are limited reports regarding preventive methods for cervical stenosis, and an optimal strategy to avoid such complications has not been established.

To our knowledge, the present study is the first RCT on an intervention to prevent cervical stenosis after LEEP in the literature, and the findings have important clinical implications for the future. One of the objectives of this study is to determine whether manual cervical dilation at regular intervals after LEEP is effective and safe for preventing post-LEEP cervical stenosis. Another objective is to investigate the incidence of cervical stenosis in postmenopausal women after LEEP, to identify the risk factors associated with post-LEEP cervical stenosis.

## Methods

### Study design and participants

**Figure 1:**
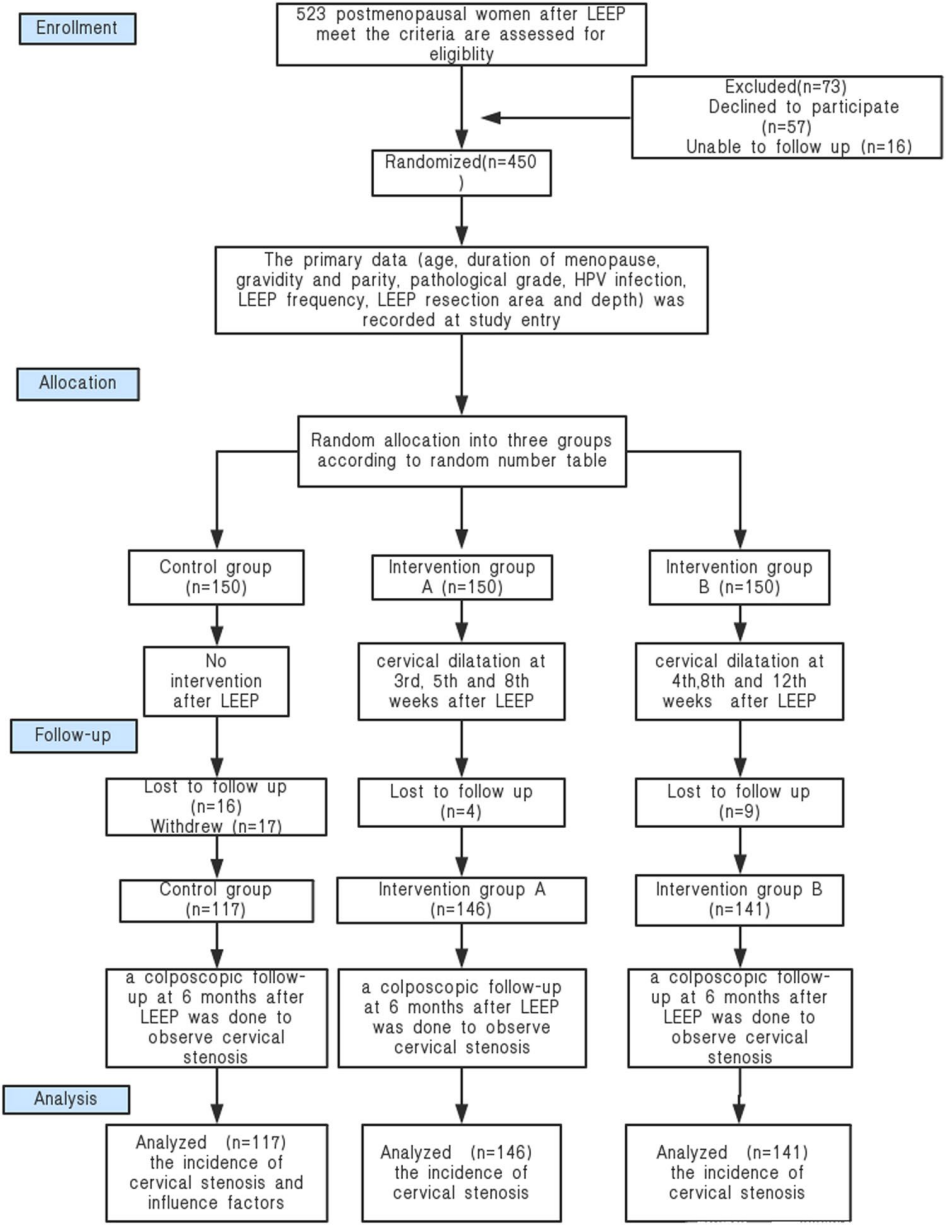
Flowchart of the inclusion and exclusion of patients during the study. Supplementary Appendix: Visual Analogue Scale.

Between August 2018 and May 2019, we conducted an RCT at the International Peace Maternity and Child Health Hospital. A flow chart of the protocol is shown in Fig. 1. Among the postmenopausal women who underwent LEEP during the study period, 450 met the inclusion criteria of the study and were examined for eligibility. Postmenopausal women who had indications for LEEP and underwent LEEP at our hospital, for whom cervical stenosis was not detected during preoperative colposcopy or LEEP were included in this study. However, we excluded non-menopausal women, patients who had cervical stenosis before LEEP, those who had not been consistently followed up, and patients with conditions such as acute inflammation of the internal and external genitals, severe heart disease, immunodeficiency disease, invasive cervical cancer, abnormal cervical anatomy, and diseases of the liver, kidney, and blood. The patients who were included were randomly allocated to three groups: patients who did not undertake any preventive measures against cervical stenosis (who were considered as the control group), and patients who used the Hegar dilator to physically dilate the cervix to prevent cervical stenosis, who were randomly allocated to intervention group A (regular cervical dilatation at the 3^rd^, 5^th^ and 8^th^ week after LEEP) and intervention group B (regular cervical dilatation at the 4^th^, 8^th^ and 12^th^ week after LEEP).

The procedure followed in this study conforms to the ethical standards set by the ethical review committee of Shanghai International Peace Maternal and Child Health Hospital, which approved of the study group. This study was reviewed and approved by the Ethics Committee of International Peace Maternal and Child Health Hospital (approval number [GKLW] 2016–62). This study has been registered with the Chinese clinical trial registry (www.clinicaltrials.gov) under registration number ChiCTR1800017629 (date of registration: 07/08/2018). All the participants provided their written informed consent for the procedures before the start of the clinical research, and the ethics committee of the hospital approved of the consent procedure. All methods in this study were strictly in accordance with the guidelines and regulations of the CONSORT guidelines for randomized controlled clinical trials.

### Randomization and masking

The eligible women (N = 404) were randomly allocated (at a ratio of 1:1:1) into three groups (control group, intervention group A and intervention group B, as mentioned earlier). A random number table generated by a computer was used for distribution and concealment. The three parts of the randomization process, namely, sequence generation, allocation concealment, and implementation, were completed by three different individuals. Because of the nature of the intervention, all participants and researchers were aware of the allocations.

### Protocol for LEEP

The patients were treated in the outpatient department. Indications for LEEP excision are as follows^5^: Unsatisfactory colposcopy (the transformation zone is not fully visualized), especially if a high-grade lesion is suspected; Suspected microinvasion; Lack of correlation between the cytology and colposcopy/biopsies, especially if a high grade lesion is suspected; Lesion extending into the endocervical canal; Endocervical curettage showing CIN or glandular abnormality; Suspected adenocarcinoma *in situ*; Colposcopist unable to rule out invasive disease; Recurrence after an ablative or previous excisional procedure.

The procedure was performed with the patients under local anesthesia; colposcopy was used for guidance and a local suction device was used to exhaust smoke. A vaginal speculum was used to expose the cervix, and 5% acetic acid and Lugol’s iodine solution were used to locate the outer edge of the cervical lesions. Then, 3–5 ml of 2% lidocaine was injected into the cervical stroma adjacent to the transformation zone in a ring pattern. All excisions were performed under strict colposcopic guidance with the help of a 1.5- to 2.0-cm wire loop electrode and a high-frequency electrical generator. The size of the loop was based on the size and distribution of lesions. The wound bed was coagulated with a ball electrode, and the 12 o’clock position in the excised specimen was marked by cutting. All the procedures were performed by an experienced gynecologist.

### Cervical dilatation strategies

Cervical dilatation was performed in all cases by the same gynecologist. In both the intervention groups (A and B), the cervix was exposed with a dilator after routine disinfection and after careful examination of the uterine axis: first, a pair of cervical forceps were used to pull the cervix into position, after which it was gradually expanded with a Hegar cervical dilator. According to the actual degree of cervical stenosis, the dilator with the size range of 2.5–4.5 mm were used in turn to mechanically dilate the cervix.. Various studies show that the average cervical length after LEEP varies from 24 to 31 mm^6,7^. According to research, cervical curettage requires a depth of at least 15 mm into the cervical canal^8^. In this study, therefore, the cervical dilatation depth after LEEP was required to reach 2 cm to make the colposcopy follow-up satisfactory. The intervention should be performed with care, and the dilation should conform to the direction of the cervical canal. Applying lubricant to the dilators can reduce friction and help to get into the cervical canal. Too much force should be avoided, and the procedure should be discontinued if apparent resistance is noted so as prevent the creation of a cervical pseudocanal and damage to the surrounding organs. After cervical dilatation, the patients were observed for 30 minutes before being released home. The participants were asked to rate pain according to 10-cm visual analog scales (VAS) from 0 (no pain) to 10 (worst possible pain). Pain intensity was measured by VAS [See Appendix for details]. The degree of pain score was defined as no pain (0), mild pain (1–4), moderate pain (5–6), or severe pain (greater than or equal to 7). Pain was assessed immediately after the procedure of cervical dilatation.

### Detection of High-risk HPV Infection

High-risk HPV (HR-HPV) was genotyped by PCR with the Cobas 4800 detection system and the corresponding reagents (Roche, Basel, Switzerland), which can detect fourteen types of HR-HPV (HPV16, 18, 31, 33, 35, 39, 45, 51, 52, 56, 58, 59, 66 and 68) and classifies HR-HPV as HPV16, HPV18, or one of 12 other types. Samples containing one or more types were considered to be positive for HR-HPV (300 and 600 copies/mL, respectively, for HPV16 and 18). The total HR-HPV load was not determined.

### Define criterion for cervical stenosis

Cervical stenosis is defined differently in different literatures. Cytological examination and colposcopy after LEEP are the keys to follow-up. This policy, however, relies on colposcopic visualization and histological confirmation of the lesion, which cannot be undertaken in women with unsatisfactory colposcopy. The identification of a transformation zone type 3 (when SCJ was not seen in its entirety) and the inability to provide a histological selection for treatment may deter cytological follow-up. Therefore, in this study, cervical stenosis was defined as the inability to visualize the entire transformation zone in colposcopy and the inability of a cervical curettor or cell brush to enter the cervical canal. It was quantified as cervical orifice less than 2.5 mm in diameter^9^ (i.e., cannot pass the 2.5 mm probe and 3 mm cell brush or cervical curettor).

### Follow-up strategy

After LEEP, the specimens obtained were fixed with 10% formaldehyde and sent to the pathology department for histological examination. The pathological findings were assessed by two senior pathologists. According to the process of wound repair and histopathological characteristics, we identified two-time points that are ideal for intervention. Accordingly, as mentioned earlier, the patients in the intervention group were instructed to return to the hospital for postoperative follow-up at the 3^rd^, 5^th^ and 8^th^ week after LEEP (intervention group A) or the 4^th^, 8^th^ and 12^th^ week after LEEP (intervention group B), which included observing wound healing, performing cervical dilatation, and recording possible complications such as genital tract infection and bleeding. However, the control group did not take the intervention measures of cervical dilation, but only observed the wound surface and recorded the complications regularly every month after LEEP. A postoperative period of 6 months is considered to be sufficient for assessing the outcome of squamocolumnar junction (SCJ) localization and the occurrence of stenosis^1^. Therefore, the subjects were followed up by colposcopic examination at 6 months after LEEP to observe the occurrence of cervical stenosis. In this study, the main outcome was the incidence of cervical stenosis after LEEP. The secondary outcomes included the different stenosis rates among different interventions and independent risk factors for cervical stenosis.

### Data analysis

To identify the indicators of cervical stenosis, univariate analysis was conducted on factors such as age, duration of menopause, gravidity and parity, pathological grade, HPV infection, LEEP frequency, LEEP resection area, and depth. The factors that were found to be significantly associated with post-LEEP cervical stenosis were used in multivariate logistic regression analysis to identify independent risk factors for stenosis. Also, differences in the incidence of clinical stenosis between the control group and the intervention groups were analyzed and compared. Quantitative data are expressed as mean and standard deviation and compared by independent *t*-tests between two samples. Qualitative data were analyzed with the chi-square test (Χ^2^ test). Logistic and multiple regression analyses were used to calculate the odds ratios to determine the risk factors for cervical stenosis. The receiver-operating characteristic curve and Youden’s index were used to calculate the cutoff of predictive indicators, for which the significance level was set at a = 0.05 (bilateral). SPSS 21.0 (IBM Corp., Armonk, NY, USA) was used for all the statistical analyses. P < 0.05 was considered to indicate statistical significance. In this study, the statistical power calculated under the current sample size is 0.92, indicating that the sample size is sufficient.

## Results

### Patient characteristics

Of the 450 women who met the study criteria, 17 women withdrew and 29 women lost to follow up after randomization. Therefore, 404 cases were finally included in this study: 117 in the control group, 146 in intervention group A and 141 in intervention group B. The average age of the participants was 58.7 ± 5.7 years, and the average menopausal duration was 7.9 ± 1.6 years. Further, the mean gravidity was 2.5 ± 1.2, and the mean parity was 1.4 ± 0.6. Concerning the clinical variables, 136 (33.7%) patients had low-grade lesions and 268 (66.3%) had high-grade lesions; further, 350 (86.6%) patients were positive for HR-HPV infection, and 54 (13.4%) did not have an HPV infection. Finally, the mean LEEP resection area was 4.2 ± 1.8 cm^2^, and the mean depth was 1.4 ± 0.3 cm. As shown in Table 1, between the intervention groups and the control group, there was no significant difference in age, duration of menopause, gravidity and parity, lesion grade, the incidence of HPV infection, LEEP frequency, and resection area and depth (P > 0.05). Similar findings were obtained when intervention groups A and B were compared (P > 0.05). Thus, the baseline characteristics of the three groups were comparable, and therefore, the patient population was suitable for random grouping.

**Table 1:** Demographic and clinical characteristics of the study population (N = 404).

	Control group (n = 117)	Intervention group(A) (n = 146)	Intervention group (B) (n = 141)	P*
Age (y)	58.34 ± 5.09	59.09 ± 5.94	58.45 ± 5.98	NS
Duration of menopause (y)	7.74 ± 2.76	8.33 ± 2.76	7.65 ± 2.97	NS
Gravidity	2.69 ± 1.29	2.47 ± 1.26	2.50 ± 0.96	NS
Parity	1.45 ± 0.54	1.30 ± 0.26	1.33 ± 0.54	NS
HR-HPV infection	81.2%	85.8%	92.3%	NS
High grade CIN	66.7%	70.2%	65.0%	NS
History of LEEP treatment	13.7%	8.5%	12.0%	NS
LEEP resection area (mm^2^)	420.22 ± 28.94	420.28± 23.97	477.94 ± 29.49	NS
LEEP resection depth (mm)	14.65 ± 2.88	14.19 ± 3.20	14.48 ± 2.99	NS

*There were no significant among group differences with regard to the characteristics at baseline.

### Incidence of cervical stenosis and complications after dilatation

All the participants were followed up for 6 months after the procedure to observe and record the occurrence of cervical stenosis. The incidence of cervical stenosis in the control group was 39.3%, and that in the intervention groups was 18.5%. The cervical stenosis rate was significantly different between the groups (P < 0.001, chi-square value = 19.53). This indicates that regular cervical dilatation can effectively reduce the rate of cervical stenosis in postmenopausal women after LEEP. Based on the VAS score, our study confirmed that cervical dilatation cause tolerable pain, 350 cases with no pain (86.7%) and 54 cases with mild pain (13.3%), during the procedure, and very mild or inexistent pain after completion of the procedure. About the complications associated with the intervention, there was only one case of bleeding in intervention group A: a 54-year-old woman reported bleeding (volume, 10 ml) on the second day of cervical dilatation, in the 3rd week after LEEP. The reason for the bleeding was found to decrustation on the wound, and hemostasis was successfully achieved by gauze compression. No other complications, such as abnormal bleeding, infection and organ injury, were observed in the intervention group within the postoperative 6 months.

Among the 99 patients with cervical stenosis, 8 of the LEEP specimens were surgical margin involvement, 5 of them were low-grade lesions and 3 of them were high-grade lesions. Among the 3 patients with high-grade lesions, 1 patient underwent hysterectomy 8 months after LEEP, and the postoperative pathology showed cervical cancer; the other 2 patients underwent LEEP again, and the pathological results showed high-grade lesions and normal pathology respectively. This evidence underlined the risk of disregard cervical neoplasms in patients with cervical stenosis.

### Effect of intervention time on the incidence of cervical stenosis

The rate of cervical stenosis was compared between group A (cervical dilatation at the 3^rd^, 5^th,^ and 8^th^ week after LEEP) and group B (cervical dilatation at the 4^th^, 8^th^ and 12^th^ week after LEEP). The rate of cervical stenosis in group A was 9.6%, and that in group B was 27.7%. The incidence was significantly different between the two groups (P < 0.001, chi-square value = 32.19). The findings indicate that regular dilation of the cervix at the 3^rd^, 5^th^ and 8^th^ week after LEEP can significantly reduce the rate of cervical stenosis.

### Risk factors for cervical stenosis

Single-factor analysis of the incidence of cervical stenosis in the control group indicated that the occurrence of cervical stenosis was significantly associated with LEEP frequency, lesion grade, resection area and depth (P < 0.05), but it was not associated with age, duration of menopause, gravidity, and parity, or presence of HPV infection (P > 0.05) (Table 2). These four independent variables identified by single-factor analysis (LEEP frequency, lesion grade, resection area, and depth) were used for multiple logistic regression analysis with cervical stenosis as the dependent variable. As shown in Table 3, the results indicated that only the frequency of LEEP and the depth of resection were independent risk factors for cervical stenosis. The predicted threshold was then calculated according to the receiver-operating characteristic (ROC) curve. When the resection depth was 16.5 mm, the maximum Youden index was 0.393. This implies that in postmenopausal women when the LEEP resection depth is greater than 16.5 mm or the frequency of LEEP is greater than 1, the incidence of cervical stenosis is higher.

**Table 2:** Univariate analysis of cervical stenosis and clinical indicators in the control group.

	No cervical stenosis (n = 56)	Cervical stenosis (n = 61)	P	OR	95% confidence interval	
					Lower limit	Upper limit
Age (y)	58.85 ± 4.91	57.48 ± 5.24	0.245	0.952	0.876	1.034
Duration of menopause (y)	7.89 ± 0.84	7.50 ± 0.68	0.823	1.010	0.925	1.103
Gravidity	2.51 ± 1.11	2.96 ± 1.48	0.277	1.146	0.897	1.463
Parity	1.34 ± 0.69	1.61 ± 0.81	0.298	1.268	0.810	1.986
HR-HPV infection (%)	83.9%	78.7%	0.469	0.707	0.276	1.811
High grade CIN (%)	57.7%	76.1%	0.042	2.300	1.047	5.054
History of LEEP treatment (%)	7.0%	19.6%	0.041	3.184	0.962	10.539
LEEP resection area (mm^2^)	377.35 ± 27.24	486.39 ± 30.50	0.046	1.196	1.041	1.374
LEEP resection depth (mm)	14.10 ± 2.58	15.50 ± 3.13	0.001	1.524	1.266	1.835

**Table 3:** Logistic regression analysis of cervical stenosis and risk factors (identified by univariate analysis) in the control group.

	B	Standard error	Walds	P	OR	95% confidence interval	
						Lower limit	Upper limit
LEEP frequency	2.292	0.382	35.994	0.000	9.893	4.679	20.915
Pathological grade	0.249	0.287	0.749	0.387	1.282	0.730	2.251
LEEP resection area	0.036	0.047	0.572	0.449	1.036	0.945	1.136
LEEP resection depth	0.125	0.045	7.856	0.005	1.133	1.038	1.236
Constant	−3.870	0.761	25.862	0.000	0.021		

### Comment

#### Principal findings

This study demonstrates the safety and effectiveness of regular cervical dilatation after LEEP as a means of preventing cervical stenosis in postmenopausal women who have undergone LEEP. Further, undergoing more than one session of LEEP and a resection depth of more than 16.5 mm were found to be risk factors for cervical stenosis in this group of patients.

## Discussion

According to previous clinical reports, menopausal transition and postmenopause and lactation periods are considered to be high-risk periods for cervical stenosis^1,10^. Besides, amenorrhea and decreased menstrual frequency have also been associated with cervical stenosis^11^. Additionally, according to a prospective study by Houlard^12^, age is a significant independent predictor of cervical stenosis after conization. However, in the present study, age was not found to be significantly associated with the risk of cervical stenosis. This is probably because all the subjects included in this study were postmenopausal women, the influence of age was not obvious.

According to Burgmann’s research^3^, a history of multiple cervical operations and a large volume of conization may increase the risk of cervical stenosis. Further, Houlard^11^ and Baldauf^13^ *et al*. reported that cervical stenosis occurred when the depth of excision was more than 20 mm, and Luesley^14^ *et al*. pointed out that greater conization depth (more than 2.5 cm) was associated with cervical stenosis. They recommended that the depth of excision be determined based on the size of the cone, thus reducing the complications. However, these studies are based on the general population, and no threshold of resection depth has been recommended for postmenopausal women. This study is the first to report the association of resection depth with the occurrence of post-procedural cervical stenosis in postmenopausal women. Based on the present findings, the threshold for resection was suggested to be 16.5 mm in postmenopausal women undergoing LEEP. It is also important to note that postmenopausal women should strictly follow the LEEP indications because the best way to reduce complications such as cervical stenosis is to avoid overtreatment.

Currently, there are no effective and reliable measures for the prevention of cervical stenosis after conization procedures. Koyama *et al*.^15^ reported a case of complete cervical stenosis caused by conization during breastfeeding and subsequent reconstruction through recanalization and dilatation of the cervical canal. Other methods reported are the temporary placement of certain types of stents and hormone replacement therapy^1,11,16^. However, Lacey *et al*.^17^ and other researchers^18,19^ have reported that estrogen replacement therapy may result in HPV infection and even HPV-associated carcinogenesis. Further, stent placement or the placement of other intrauterine devices is limited by their low feasibility, high cost, and risk of infection. In the present study, we have explored the use of a manual cervical dilatation strategy with a Hegar dilator as a means of preventing cervical stenosis: the incidence of cervical dilatation in the intervention groups was significantly lower than that in the control group. Moreover, bleeding occurred in only one of the patients, who was immediately treated.

It is important to note that a single cervical dilation is only effective temporarily, and there is a risk of cervical stenosis occurring at a later time point^9^. Currently, there is no standard dilatation procedure or frequency recommended. Therefore, in the present study, we also compared the effect of the frequency of cervical dilatation after LEEP. Based on published information about the wound healing process, we determined that the 3^rd^ week after LEEP was an ideal time point to start the dilatation intervention. Accordingly, the intervention time points were the 3^rd^, 5^th^ and 8^th^ week after LEEP in the first group and the 4^th^, 8^th^ and 12^th^ week after LEEP in the other group. The findings clearly showed that the incidence of cervical stenosis was significantly lower in the former group.

### Clinical implications

Cervical stenosis might be easier to diagnose in women of childbearing age, as it might be accompanied by obvious clinical symptoms such as dysmenorrhea, amenorrhea, hematoma, pyometra, pelvic pain, and infertility^20^. However, in postmenopausal women, there is often no obvious clinical manifestation, and hence, it is easy to miss this condition. At every stage of a woman’s life, a satisfactory follow-up is the main requirement for the conservative management of CIN. Tanaka *et al*. found that in elderly patients with cervical stenosis, the recurrence rate was higher if the surgical margin involvement was also present^21^. Follow-up with screening cytology or biopsy should be considered in younger patients. However, in those older patients with cervical stenosis, hysterectomy may be a reasonable option due to the majority of patients with cervical stenosis resulting in inadequate cancer screening^22^. Therefore, the findings of this study are important, as they focus on follow-up interventions to prevent cervical stenosis in postmenopausal women who have undergone LEEP, and also highlight the frequency of LEEP and resection depth as significant risk factors that can be used to screen postmenopausal women.

Here, we have recommended the use of a Hegar dilator at the 3^rd^, 5^th^ and 8^th^ weeks after LEEP. The Hegar dilator is readily available around the world, so most women would have easy access to this method. Further, the type and diameter of the rod can be adjusted according to the patient’s need. An added advantage is that this method does not require the patient to be under anesthesia and can be easily performed in outpatient clinics. Finally, the use of the dilator was not associated with any major complications.

### Strengths and limitations

The intervention strategy proposed in this study could significantly reduce the incidence of cervical stenosis after LEEP in postmenopausal women, and it was found to be highly effective and safe. This study is the first RCT on an intervention to prevent cervical stenosis after LEEP in the literature, and the findings have important clinical implications for the future. It lays the ground for large-scale, multi-center RCTs for verifying the practicality and effectiveness of this intervention. The study’s main limitation is that it is a single-center study with a small sample size. Further, considering the limited follow-up time of 6 months and the preventive effect of topical estrogen therapy on the occurrence of cervical stenosis, we are preparing a follow-up study to include more patients in our clinical population, extend follow-up time, and consider topical estriol treatment to reduce cervicovaginal dystrophy. We would like to see colleagues conduct confirmatory and even prospective studies in multiple centers. Identifying and promoting these findings to different institutions and clinic settings can even call for changes in the management of postmenopausal LEEP patients.

## Conclusions

Regular manual dilatation of the cervix with a Hegar dilator after LEEP can be recommended as a reliable and safe intervention to prevent cervical stenosis in postmenopausal women, with the 3^rd^, 5^th^ and 8^th^ weeks after LEEP being optimal time points. Finally, the frequency of LEEP and the depth of resection are implicated as independent risk factors for cervical stenosis in postmenopausal women.

## Supplementary information

Supplementary Information.
