# Supplementary material for: A new approach to prevent cervical stenosis in postmenopausal women after loop electrosurgical excision procedure: a randomized controlled trial
Source: Sci Rep. 2020 May 22;10:8512. doi: 10.1038/s41598-020-65170-2 (PMC7244737; doi:10.1038/s41598-020-65170-2)

**Visual Analogue Scale**

**Name______________ Date_______________**

**Patient ID___________**

**If “0” is “no pain” and “10” is the worst pain you can imagine. Where is your pain now? Place a mark on the line blow to show the amount of pain that you feel.**


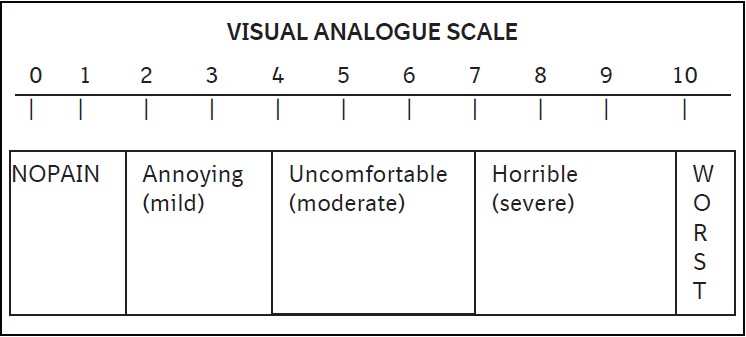

Supplement: Supplementary file 1 — Supplementary Information. [file 41598_2020_65170_MOESM1_ESM.doc]
